# Linking Microbiota Profiles to Disease Characterization in Common Variable Immunodeficiency: The Case of Granulomatous–Lymphocytic Interstitial Lung Disease

**DOI:** 10.3390/biomedicines12102239

**Published:** 2024-10-01

**Authors:** Marta Dafne Cabanero-Navalon, Miguel Carda-Diéguez, Pedro Moral Moral, Alex Mira, Héctor Balastegui-Martin, Miguel Salavert-Lletí, Victor Garcia-Bustos

**Affiliations:** 1 Primary Immunodeficiencies Unit, Department of Internal Medicine, University and Polytechnic Hospital La Fe, 46026 Valencia, Spain; moral_ped@gva.es (P.M.M.); balastegui_hec@gva.es (H.B.-M.); garcia_vicbus@gva.es (V.G.-B.); 2 Research Group of Chronic Diseases and HIV Infection, Health Research Institute La Fe, 46026 Valencia, Spain; 3 Genomics & Health Department, FISABIO Foundation, 46020 Valencia, Spain; miguel.carda@uv.es (M.C.-D.); alex.mira@fisabio.es (A.M.); 4 Unit of Infectious Diseases, Department of Internal Medicine, University and Polytechnic Hospital La Fe, 46026 Valencia, Spain; salavert_mig@gva.es; 5 Severe Infection Research Group, Health Research Institute La Fe, 46026 Valencia, Spain

**Keywords:** common variable immunodeficiency, granulomatous–lymphocytic interstitial lung diseases, microbiome profile, immune dysregulation, microbiota

## Abstract

**Background and objectives:** Common variable immunodeficiency (CVID) is a primary immunodeficiency characterized by decreased immunoglobulins and recurrent infections, with non-infectious complications such as granulomatous–lymphocytic interstitial lung disease (GLILD) affecting up to 30% of patients. **Methods:** Using high-throughput 16S rRNA gene sequencing, salivary, sputum, and fecal microbiome from CVID patients with GLILD, comparing them to CVID patients without GLILD—with immune dysregulation (dCVID) and only infections (iCVID)—and healthy controls was analyzed. **Results:** A total of 41 CVID patients, 7 with GLILD, and 15 healthy donors were included. Global fecal biodiversity was significantly lower in GLILD patients compared to CVID subgroups and controls. GLILD patients harbored different specific bacterial communities in all niches, with some keystone species common to dCVID. *Conchiformibius*, *Micrococcales*, and *Capnocytophaga* are more frequent in the sputum of GLILD patients. Saliva in GLILD shows higher frequencies of *Conchiformibius* and *Haemophilus* *parainfluenzae*. Fecal samples from GLILD patients have higher levels of *Gemella morbilorum*, *Lacticaseibacillus*, and *Cellulosimicrobium*. A non-assigned *Conchiformibius* spp. is consistently associated with GLILD across different niches and could be a potential pathobiont or relevant microbiological marker for GLILD. Cluster network and correlation analyses show profound dysbiosis in the sputum, saliva, and feces of GLILD patients. **Conclusions:** These findings highlight significant microbiome alterations in CVID patients with GLILD, particularly in the respiratory tract, suggesting a possible link to both local and systemic immune dysregulation.

## 1. Introduction

Common variable immunodeficiency (CVID) is the most common symptomatic primary immunodeficiency (PID), characterized by decreased immunoglobulins and recurrent infections after ruling out secondary causes of hypogammaglobulinemia [1]. Historically, infections were the main cause of morbidity and mortality among CVID patients until the introduction of immunoglobulin replacement therapy (IgRT) in the late 20th century. Non-infectious complications such as autoimmunity, benign lymphoproliferative disorders, and neoplasia [2,3] have emerged as the comorbidities with a larger impact on prognosis and quality of life over infections, involving up to 70% of patients [4,5,6]. In addition, granulomatous–lymphocytic interstitial lung disease (GLILD) is a non-infectious lung complication that develops in 9% to 30% of patients with CVID [7,8] and has been associated with long-term lung damage and poorer clinical outcomes in symptomatic patients [9]. However, the pathophysiology underlying this immune dysregulation-derived manifestation as well as the mechanisms influencing its development in specific subsets of CVID patients remain poorly understood.

It is believed that multiple genetic and environmental risk factors interact to contribute to these disorders [10]. Several studies have primarily focused on investigating the intestinal microbiome in patients with CVID, given that the majority of the human microbiome is located in the gastrointestinal (GI) tract. Data on other microbial niches, such as saliva or sputum, remain limited. This is particularly crucial in conditions like GLILD, where lung microbiota-driven immune dysregulation may play a key pathophysiological role.

In CVID, impaired immunity may lead to increased microbial translocation across the gut barrier, triggering persistent systemic immune activation. This chronic immune activation could drive immune dysregulation, potentially contributing to the non-infectious complications commonly observed in CVID patients [11,12]. Elevated serum levels of lipopolysaccharide (LPS) and immune markers, such as soluble CD14 and IL-2, have been observed in patients with CVID and have been correlated with reduced alpha diversity and a higher dysbiosis index in their gut microbiota. Notably, these changes were more pronounced in CVID patients with inflammatory and autoimmune complications compared to those with only infectious complications [11].

Furthermore, the lung microbiota is being investigated as a potential cause of local immune dysregulation and lymphoproliferation, similar to what is observed in other systemic autoimmune diseases like sarcoidosis and interstitial lung diseases (ILD) [13,14]. This hypothesis could potentially be extended to patients with GLILD. Moreover, recent studies propose a possible bidirectional link between gut and lung microbiota, referred to as the gut–lung axis [15,16]. Additionally, alterations in the oropharyngeal microbiota have been documented in patients with ILD and primary antibody deficiencies [17,18], such as GLILD in CVID, reflecting modifications in the pulmonary microbiota due to bacterial seeding from the lower respiratory tract.

Our group has demonstrated distinct microbiota profiles in the lungs, saliva, and feces of CVID patients, correlating with clinical phenotypes and associated immune dysregulation complications [19]. However, the specific composition of microbiota in the lung, saliva, and feces of GLILD patients has not yet been analyzed and distinguished from the rest of CVID patients or the healthy population. This is of crucial importance, as this complication is very frequently associated with systemic immune dysregulation symptoms such as splenomegaly or benign lymphoproliferation, and many authors suggest that GLILD may represent the pulmonary manifestation of a broader systemic disease [6].

Hence, we aim to provide further insights into disease pathogenesis and expand the limited evidence regarding microbiota in CVID patients, with a specific focus on those with GLILD. Specifically, we seek to determine if their saliva, lung, and fecal microbiota profiles differ from those of other CVID patients and to identify potential pathobionts more closely associated with local immune dysregulation and lymphoproliferation in the lung, which could drive the development of GLILD.

## 2. Materials and Methods

### 2.1. Patients

A cross-sectional study was conducted, including 41 patients diagnosed with CVID according to the European Society for Immunodeficiencies (ESID) criteria, aged over 18 years, and followed in the PID Unit of the Department of Internal Medicine at the University and Polytechnic Hospital La Fe. Demographic, clinical, and laboratory parameters were retrospectively investigated for all patients, following the methodology of Cabanero et al. [19].

Patients were stratified into three groups based on clinical parameters according to the classification by Chapel et al. [5] and the presence of GLILD. The first group included CVID patients with GLILD, confirmed by chest high-resolution CT (HRCT), bronchoalveolar lavage excluding infectious pneumonia, and histological confirmation via lung biopsy using either video-assisted thoracoscopic surgery (VATS) or transbronchial biopsy, excluding malignancy. The second group consisted of CVID patients with a history of immune dysregulation, manifested by autoimmune hemolytic anemia (AHA), immune thrombocytopenic purpura (ITP), Evans’ syndrome, non-infectious lymphadenopathies, hepatopathy, splenomegaly, non-infectious chronic enteropathy, and/or solid or hematologic malignancies, classified as ‘dysimmune CVID or CVID with immune dysregulation’ (dCVID), excluding those with GLILD as previously defined. The third group comprised CVID patients without immune dysregulation-related complications and had only developed infections, classified as ‘infectious-only CVID’ (iCVID).

**Figure 1:**
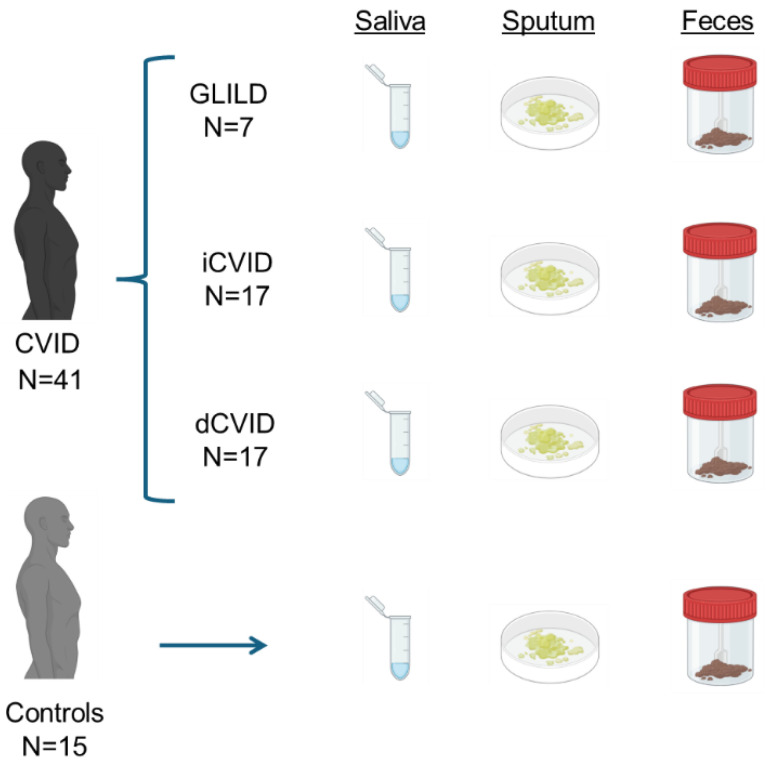
Description of the study design. GLILD: granulomatous–lymphocytic interstitial lung disease. dCVID: common variable immunodeficiency with immune dysregulation. iCVID: common variable immunodeficiency with only infections.

In addition, 15 healthy donors, unrelated to the previously mentioned patients, were also included in the study. Their medical histories were reviewed for the presence of any of the listed pathologies, and if such conditions were present, these donors were excluded from the study. The stratification of participants can be viewed in Figure 1. 

This study was approved by the Ethical Committee of Health Research Institute La Fe with registry code 2020-376-1 and was conducted in accordance with the Declaration of Helsinki.

### 2.2. Sampling and DNA Extraction

In this study, oral, sputum, and fecal samples were collected from 41 CVID patients and 15 healthy donors. Controls did not have active caries or periodontal disease. The use of immunosuppressive therapy, prophylactic antibiotics, and probiotics was documented. Patients who had received prophylactic antibiotics other than cotrimoxazole or azithromycin in the past month were excluded, as were healthy donors who had taken antibiotics within one month before sampling, to minimize the impact of antibiotic use on microbiota composition [20].

Saliva samples (1 mL) were collected after three minutes of unstimulated salivation. Sputum samples (1 mL) were collected after deep breathing, followed by a productive cough. For fecal sampling, 5 mL of feces was collected in a flask with RNAlater^®^, kept at room temperature until laboratory delivery, and then stored at −80 °C until DNA extraction.

DNA was extracted using the MagNa Pure LC DNA Isolation Kit II and MagNa Pure Instrument (Roche, Mannheim, Germany). Samples underwent ultrasound lysis (three 10 s cycles) and enzymatic digestion with lysostaphin, lysozyme, and mutanolysin, followed by Proteinase K degradation [21]. The V3-V4 hypervariable regions of the 16S rRNA gene were amplified using optimized universal primers for Illumina sequencing [22]. High-throughput sequencing was performed using Illumina Miseq (Illumina, Inc., San Diego, CA, USA), with library construction following the 16S rRNA gene Metagenomic Sequencing Library Preparation Illumina protocol (Part #15,044,223 Rev. A). Sequencing was conducted at the FISABIO Institute using the 2 × 300 bp paired-end Illumina protocol. Further details can be found in the Materials and Methods section of [19].

### 2.3. Bioinformatic Analysis and Statistics

Clinical characteristics of the participants were compared using Fisher’s exact test and ANOVA after verifying normality with Q–Q plots and variance equality with Levene’s test. For the microbiome analysis, Dada2 (v1.16) software was used to filter, end-trim, denoise, and merge paired reads [23]. Adapters and primers were removed, and sequences were end-trimmed in 10 bp windows with no Ns and quality values above 35. Singletons were removed except for richness and diversity index calculations. The filtered reads were merged, clustered, and cleaned for host and chimeric reads. High-quality sequences were then processed through the Dada2 pipeline and assigned at the amplicon sequence variants (ASV) and species levels using the SILVA database v138.1 [24]. 

The R programming language was used for downstream analysis. Genera with abundances lower than 0.01% were excluded. Multivariate analyses were performed using the Adonis test from the Vegan library in R [25]. The 16S data were normalized and compared using the Analysis of Composition of Microbiomes with Bias Correction (ANCOM-BC) test. Diversity indexes were compared using the Wilcoxon test. Taxa with abundances smaller than the closest value to zero multiplied by four in less than 60% of samples were removed from the ANCOM-BC comparison. Additionally, correlations among bacterial taxa were assessed using Spearman’s rank correlation method. Bacterial abundances were normalized using the ANCOM-BC2 approach and filtered to include only those with an abundance greater than seven times the lowest value close to zero and present in ≥60% of samples. Correlations with R ≥ 0.7 were considered for interpretation. Network and heatmap visualizations were generated using the mixOmics package in R (version 6.1.3) [26,27]. Further details are available in the Materials and Methods section of Cabanero et al., 2023 [19].

## 3. Results

### 3.1. Population Characteristics

A total of 41 CVID patients and 15 healthy controls were sampled. Among CVID patients, 7 were classified as GLILD, 17 as dCVID, and 17 as iCVID. The mean ages were 36.71 (SD 17.13) for GLILD patients, 46.82 (SD 14.91) for dCVID patients, 49.88 (SD 16.90) for iCVID patients, and 44.49 (SD 14.19) for healthy controls. No significant differences were found. The male sex distribution was as follows: 3 GLILD patients (42.9%), 9 dCVID patients (52.9%), 5 iCVID patients (29.4%), and 6 healthy controls (40%). No statistically significant differences were observed. The primary comorbidities in GLILD patients included lymphadenopathies (85.7%), splenomegaly (71.4%), autoimmune hemolytic anemia (42.9%), immune thrombocytopenic purpura (42.9%), Evans’ syndrome (14.3%), and non-infectious immune enteropathy (14.3%). The Baumann-GLILD score was calculated for GLILD patients, with a mean of 18.5 (SD 4.86). No patients with GLILD had a history of malignancy. One patient showed a heterozygotic mutation in the PI3KR1 gene of uncertain significance (c.5A > T p.Tyr2Phe). 

History of immunosuppressant treatment was recorded in 4 GLILD patients (57.1%), 5 dCVID patients (29.4%), and 1 iCVID (5.9%) (Fisher *p* = 0.027). History of prophylactic antibiotic therapy was noted in 4 GLILD patients (57.1%), 3 dCVID patients (17.6%), and 2 iCVID (11.8%).

### 3.2. Global Microbiome Biodiversity Indicators

**Figure 2:**
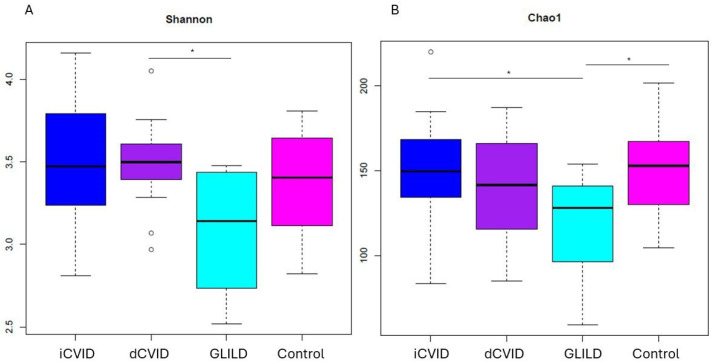
Shannon (**A**) and Chao1 (**B**) alpha diversity indices of fecal microbiota in GLILD patients, CVID groups, and healthy controls. dCVID: common variable immunodeficiency with immune dysregulation. GLILD: granulomatous–lymphocytic interstitial lung disease. iCVID: common variable immunodeficiency with only infections. Asterisks indicate statistical significance (*p* < 0.05).

No significant differences were observed in the Chao1 (richness) and Shannon (evenness) indices between GLILD patients and the rest of the CVID patients (dCVID or iCVID) or compared to healthy controls in saliva and sputum samples. However, the overall fecal biodiversity, in terms of richness and evenness measured by the Chao1 and Shannon indices, respectively, was significantly lower in GLILD patients compared to CVID patients and healthy controls (*p* < 0.05). Specifically, the richness of the fecal microbiome in GLILD patients was significantly lower compared to dCVID patients, iCVID patients, and healthy controls (*p* < 0.05). The bacterial evenness of the fecal microbiome in GLILD patients was also significantly lower than in dCVID patients (*p* < 0.05) (Figure 2).

### 3.3. Microbiological Differences in GLILD Patients, CVID Subgroups, and Healthy Controls

#### 3.3.1. Sputum

**Figure 3:**
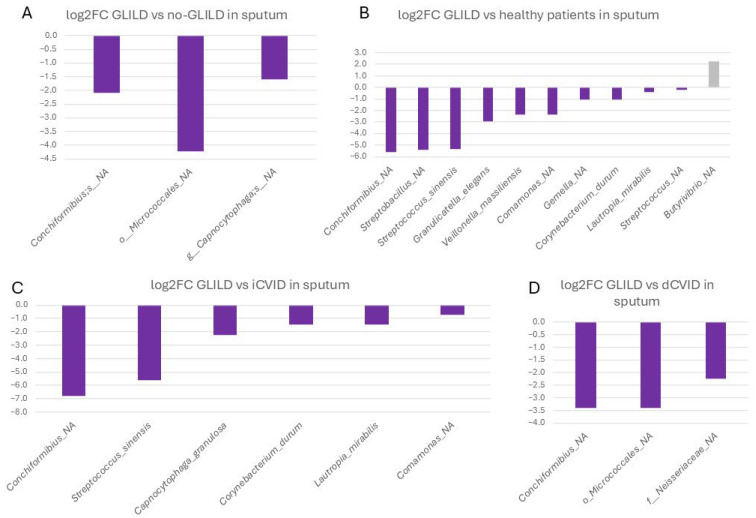
Differential microbial profiles in the sputum of GLILD patients, CVID groups, and healthy controls. (**A**) GLILD vs. no-GLILD, (**B**) GLILD vs. healthy controls, (**C**) GLILD vs. iCVID, and (**D**) GLILD vs. dCVID. Bars represent log2 fold changes in microbial abundance. Purple bars indicate microbial taxa with increased abundance in GLILD patients (negative values), while other colors represent the comparator groups, where positive values indicate increased abundance in the comparator group compared to GLILD. dCVID: common variable immunodeficiency with immune dysregulation. GLILD: granulomatous–lymphocytic interstitial lung disease. iCVID: common variable immunodeficiency with only infections. Log2FC: log2 fold change.

In sputum, an unassigned species of *Conchiformibius* spp. (0.020% vs. 0.0009%), an unassigned species of the order *Micrococcales* (0.006% vs. 0.001%), and an unassigned species of *Capnocytophaga* (0.395% vs. 0.131%) were significantly more frequent in GLILD patients compared to non-GLILD CVID patients (Figure 3).

Specifically, when comparing GLILD patients to dCVID patients, these differences remained significant for the unassigned species of *Conchiformibius* spp. (0.020% vs. 0.002%) and the unassigned species of the order *Micrococcales* (0.006% vs. 0.0004%), with the addition of an unassigned species of the family *Neisseraceae* (0.004% vs. 0.0007%).

Concerning iCVID patients, the unassigned species of *Conchiformibius* spp. continued to be more prevalent in GLILD patients (0.020% vs. 0%). Additionally, the frequencies of *Streptococcus sinensis* (0.009% vs. 0%), *Capnocytophaga granulosa* (0.146% vs. 0.031%), *Corynebacterium durum* (0.096% vs. 0.003%), *Lautropia mirabilis* (0.088% vs. 0.032%), and an unassigned species of *Comamonas* spp. (0.013% vs. 0.008%) were significantly higher in the sputum of GLILD patients.

GLILD patients exhibited significantly higher relative abundances of several bacterial species in sputum compared to healthy controls, including the same unclassified species of *Conchiformibius* (0.020% vs. 0.0002%), an unassigned species of *Streptobacillus* spp. (0.091% vs. 0.002%), *S. sinensis* (0.009% vs. 0%), *Granulicatella elegans* (1.214% vs. 0.159%), *Veillonella massiliensis* (0.113% vs. 0.021%), the same unassigned species of *Comamonas* spp. (0.014% vs. 0.002%), an unassigned species of *Gemella* spp., *C. durum, L. mirabilis*, and an unassigned species of *Streptococcus* spp. In healthy individuals, an unassigned species of *Butyrivibrio* spp. was significantly more prevalent (0.07% in healthy controls vs. 0.014%).

#### 3.3.2. Saliva

**Figure 4:**
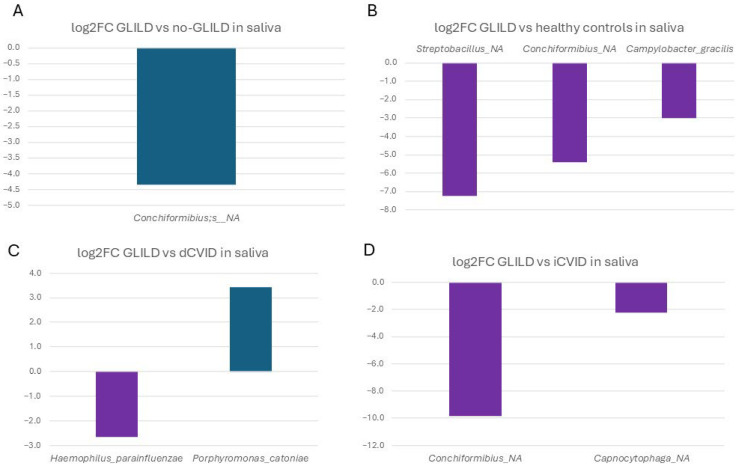
Differential microbial profiles in the saliva of GLILD patients, CVID subgroups, and healthy controls. (**A**) GLILD vs. no-GLILD, (**B**) GLILD vs. healthy controls, (**C**) GLILD vs. dCVID, and (**D**) GLILD vs. iCVID. Bars represent log2 fold changes in microbial abundance. Purple bars indicate microbial taxa with increased abundance in GLILD patients (negative values), while other colors represent the comparator groups, where positive values indicate increased abundance in the comparator group compared to GLILD. dCVID: common variable immunodeficiency with immune dysregulation. GLILD: granulomatous–lymphocytic interstitial lung disease. iCVID: common variable immunodeficiency with only infections. Log2FC: log2 fold change.

In saliva, the unassigned species of *Conchiformibius* spp. was significantly more frequent in GLILD patients compared to non-GLILD patients (0.1% vs. 0.005%) (Figure 4). When comparing GLILD patients to dCVID patients, GLILD patients had significantly higher populations of *Haemophilus parainfluenzae* (1.681% vs. 0.265%), while dCVID patients had higher frequencies of *Porphyromonas catoniae* (0.161% vs. 0.0147%).

Compared to iCVID patients, GLILD patients had significantly higher populations of the same unassigned species of *Conchiformibius* spp. (0.1% vs. 0%) and an unassigned species of *Capnocytophaga* spp. (0.937% vs. 0.196%).

Finally, compared to healthy individuals, the saliva of GLILD patients had significantly higher frequencies of an unassigned species of *Streptobacillus* (0.066% vs. 0.0003%), the unassigned species of *Conchiformibius* spp. (0.01% vs. 0.002%), and *Campylobacter gracilis* (0.094% vs. 0.011%).

#### 3.3.3. Feces

**Figure 5:**
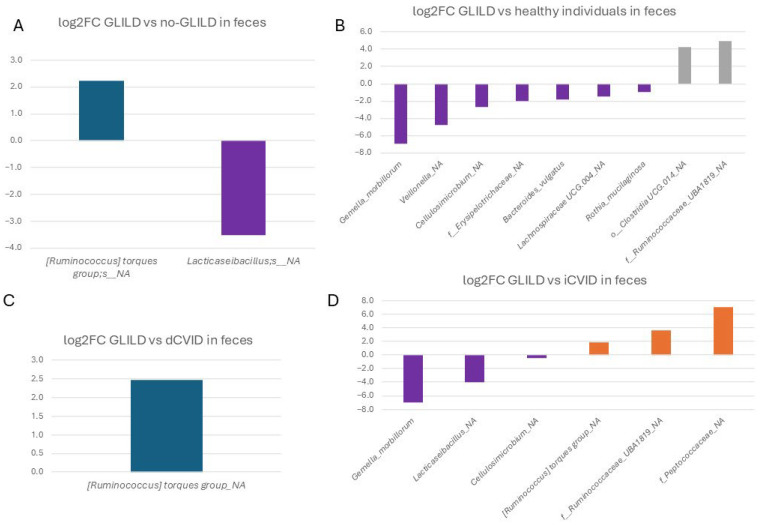
Differential microbial profiles in the feces of GLILD patients, CVID groups, and healthy controls. (**A**) GLILD vs. no-GLILD, (**B**) GLILD vs. healthy individuals, (**C**) GLILD vs. dCVID, and (**D**) GLILD vs. iCVID. Bars represent log2 fold changes in microbial abundance. Purple bars indicate microbial taxa with increased abundance in GLILD patients (negative values), while other colors represent the comparator groups, where positive values indicate increased abundance in the comparator group compared to GLILD. dCVID: common variable immunodeficiency with immune dysregulation. GLILD: granulomatous–lymphocytic interstitial lung disease. iCVID: common variable immunodeficiency with only infections. Log2FC: log2 fold change.

In feces, an unspecified species of *Ruminococcus* from the torques group was significantly more frequent in non-GLILD patients (1.25% vs. 0.267%), while an unassigned species of *Lacticaseibacillus* (0.007% vs. 0.0004%) was significantly more frequent in GLILD patients compared to non-GLILD patients (Figure 5).

Specifically, comparing GLILD patients to dCVID patients, the same unspecified species of *Ruminococcus* from the torques group was significantly more frequent in dCVID patients than in GLILD patients (0.267% vs. 0.0472%).

Compared to iCVID patients, the feces of GLILD patients exhibited significantly higher relative abundances of *Gemella morbillorum* (0.028% vs. 0%), an unassigned species of *Lacticaseibacillus* spp. (0.007% vs. 0.0002%), and an unassigned species of *Cellulosimicrobium* spp. (0.001% vs. 0.0008%). Conversely, iCVID patients had significantly higher populations of an unassigned species of *Peptococcaceae* (0.031% vs. 0%), an unassigned species of *Ruminococcaceae* UBA1819 (0.061% vs. 0.0048%), and the unspecified species of *Ruminococcus* from the torques group (0.974% vs. 0.267%).

Regarding the fecal microbiota of GLILD patients compared to healthy controls, GLILD patients had significantly higher frequencies of *G. morbilorum* (0.028% vs. 0%), an unassigned species of *Veillonella* (0.61% vs. 0.022%), an unassigned species of *Cellulosimicrobium* (0.001% vs. 0%), an unassigned species of *Erysipelotrichaceae* (0.023% vs. 0.006%), *Bacteroides vulgatus* (0.937% vs. 0.269%), an unassigned species of *Lachnospiraceae* UCG.004 (0.104% vs. 0.0367%), and *Rothia mucilaginosa* (0.003% vs. 0.001%). However, healthy controls had higher populations of an unassigned species of Clostridia UCG.014 (2.656% vs. 0.166%) and an unassigned species of *Ruminococcaceae* UBA1819 (0.153% vs. 0.0048%).

#### 3.3.4. Pathobionts, Patterns, and Species Associated with GLILD in the Different Niches

In sputum, an unassigned species of *Conchiformibius* spp. is consistently associated with GLILD, showing significant differences compared to all other groups, including the dCVID group with immune dysregulation. This pattern is also observed in saliva, where this species is significantly more frequent compared to all groups except dCVID. This species could serve as a relevant microbiological marker for GLILD.

Besides the unassigned species of *Conchiformibius*, an unassigned species of *Capnocytophaga*, *C. durum*, *L. mirabilis*, *S. sinensis*, and an unassigned species of *Comamonas* spp. are also associated with GLILD, showing significant differences compared to all other groups, although no significant differences were observed when compared to dCVID. These species may be associated with the immune dysregulation characteristic of GLILD and other CVID subgroups.

In fecal microbiota, two unassigned species of *Lacticaseibacillus* and *Cellulosimicrobium*, along with *Gemella morbilorum*, are identified as potential markers of GLILD compared to CVID groups, particularly iCVID and healthy controls. However, these species did not show significant distinction when compared to the dCVID group, where only an unassigned species of *Ruminococcus* from the torques group shows relevance, though not significant, compared to healthy individuals.

**Figure 6:**
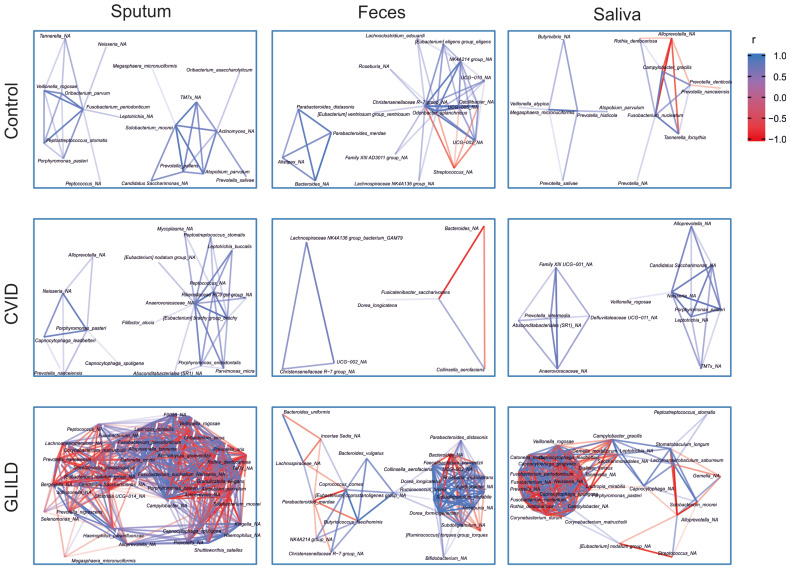
Cluster analysis of bacterial taxa in the microbiota in sputum, feces, and saliva of GLILD, CVID patients, and healthy controls. In controls, bacterial networks are sparse, with moderate interactions, particularly in sputum, while feces show fewer correlations. CVID patients exhibit more diverse bacterial interactions in sputum and saliva, with a less structured network in feces. GLILD patients display dense and complex networks, especially in sputum and feces, with a mix of positive (blue) and negative (red) correlations, indicating significant microbial dysbiosis.

**Figure 7:**
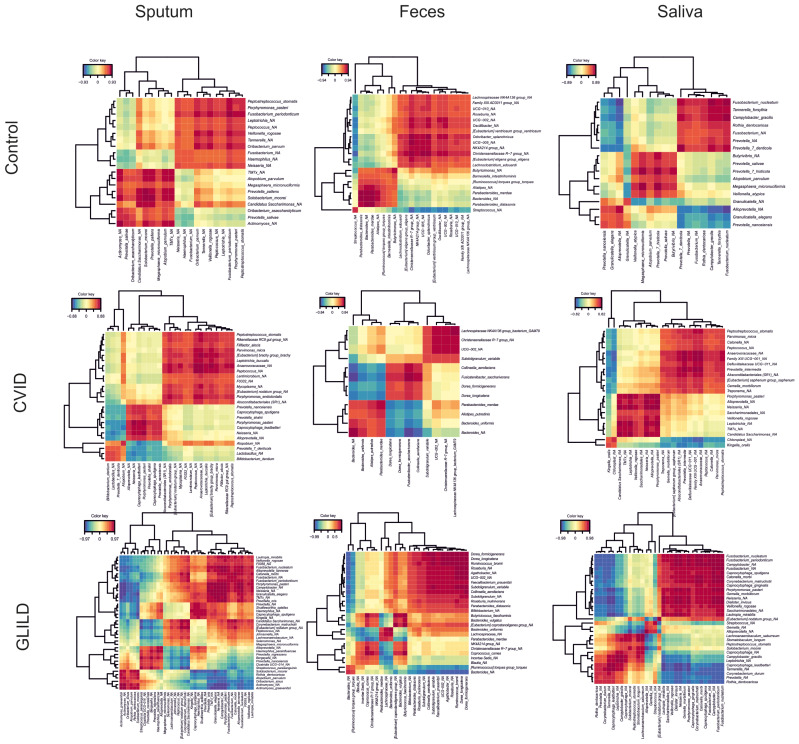
Heatmap of bacterial correlations in sputum, feces, and saliva of GLILD, CVID patients, and healthy controls. Positive correlations (red) indicate co-occurrence between bacterial taxa, while negative correlations (blue) indicate inverse relationships. In the control group, correlations are relatively uniform, with fewer strong associations, suggesting a balanced microbiome. In the CVID group, there is moderate variability, with both positive and negative correlations, particularly in sputum and saliva. The GLILD group exhibits the most disrupted correlation patterns with numerous strong positive and negative correlations, especially in feces, reflecting significant microbial dysbiosis. This suggests an increasing disruption of microbial networks from controls to GLILD patients.

Additionally, cluster network analysis illustrating bacterial correlations across the three sample types and patient groups reveals marked differences in microbiota structure between patient cohorts. GLILD patients exhibit the most disrupted and highly interconnected bacterial profiles, indicating profound bacterial dysbiosis (Figure 6). The correlation heatmap also reveals distinct microbial interaction patterns across patient groups (Figure 7). Controls show relatively balanced correlations, while CVID patients exhibit moderate variability, particularly in sputum and saliva. GLILD patients display the most disrupted microbiota, with strong positive and negative correlations, especially in feces, indicating severe microbial dysbiosis. 

## 4. Discussion

In this study, we performed the first analysis of the microbiome profile in CVID patients with GLILD, focusing on differences in global biodiversity and specific bacterial species in three niches: saliva, sputum, and feces. Using NGS techniques and advanced statistical analyses, we conducted comparative studies with CVID patients without GLILD and sex and age-paired healthy controls. Additionally, we explored potential differential microbial mechanisms of immune dysregulation by comparing GLILD patients with other CVID subgroups exhibiting immune dysregulation complications and CVID patients experiencing only infections. 

The main findings of this study can be summarized as follows: (i) Global fecal biodiversity is significantly lower in GLILD patients compared to CVID subgroups and healthy controls. (ii) GLILD patients harbor different specific bacterial communities in saliva, sputum, and feces, with some keystone species common to other CVID patients with immune complications. (iii) *Conchiformibius*, *Micrococcales*, and *Capnocytophaga* are more frequent in the sputum of GLILD patients. (iv) Saliva of GLILD patients shows higher frequencies of *Conchiformibius* and *H. parainfluenzae*. (v) Fecal samples from GLILD patients have higher levels of *Gemella morbilorum*, *Lacticaseibacillus*, and *Cellulosimicrobium.* (vi) A non-assigned *Conchiformibius spp.* is consistently associated with GLILD across different niches and could be a potential pathobiont or relevant microbiological marker for GLILD. (vii) Cluster network and correlation analyses show profound dysbiosis in the sputum, saliva, and feces of GLILD patients.

Currently, there is no evidence of the potential microbial triggers of GLILD in CVID. Nevertheless, in recent years, there has been a growing interest in characterizing the microbiome of CVID patients, especially those with immune complications [9,11,28,29,30]. Dysbiosis has been clinically associated with immune dysregulation in CVID, as well as elevated levels of sCD14, sCD25, and LPS [11,12,19] and a proinflammatory lipid profile [31]. Serum bacterial DNA levels have been correlated with systemic immune activation parameters, elevated serum IFN-γ, and lower counts of isotype-switched memory B cells, and trigger strong host IFN-γ responses in dCVID [32]. Current evidence is, however, limited, and research efforts are needed to address the underlying pathophysiological mechanisms of immune dysregulation, especially considering that mainly all studies address gut microbiota and only our group has analyzed the respiratory niche in these patients [19]. 

Several microbial markers of GLILD in our work have been previously reported as more frequent in CVID, such as *H. parainfluenzae*, *Bacteroides vulgatus, Gemella* spp. [33], *Ruminococcaceae* and *Ruminococcus* spp. [34,35], *Lactobacillales* [29], and *Streptococcus* spp. [33], which shows an increased relative abundance of ILD in humoral immunodeficiencies (Berbers et al., 2020) and CVID patients with chronic obstructive pulmonary disease (COPD) and older age [29]. However, despite some of them reporting several cases of GLILD, no information is given about their microbial profiles. 

The consistent finding of a non-assigned species of *Conchiformibius,* an aerobe, Gram-negative chemoorganotrophic bacterium, as an independent marker of GLILD, even when compared to other CVID patients with autoimmunity or immune dysregulation, is uncertain. The genus has been associated previously with autoimmunity in psoriasis patients [36], but there are no reports of CVID or respiratory diseases in humans, and it has only been identified as a biomarker of idiopathic pulmonary fibrosis in dogs [37]. In the respiratory tract, this species might influence the immune response by promoting a proinflammatory environment, possibly via interactions with epithelial cells or macrophages, leading to lung tissue remodeling or fibrosis. However, its exact mechanism remains unclear and warrants further investigation, particularly to assess its role as a pathobiont triggering chronic inflammation in GLILD.

Coincidentally, some of the bacterial biomarkers previously described in our population of dCVID patients are also present in GLILD individuals, suggesting a potentially similar distribution and behavior in these proinflammatory landscapes, such as *C. durum, L. mirabilis, Veillonella* spp.—also present in other autoimmune diseases—*C. granulosa,* or *G. elegans* [19]. 

Additionally, and as in our work, the abundance of several genera has been reported to be higher in GLILD-like diseases, such as non-CVID-related ILD and sarcoidosis, such as *Streptococcus*, *Haemophilus,* and *Veillonella* [38,39,40], *Granulicatella* in the context of sarcoidosis [38], or *Gemella* in pulmonary idiopathic fibrosis [41]. 

Interestingly, *H. parainfluenzae,* significantly more frequent in GLILD patients, is known to be part of the normal flora but has been implicated in various respiratory conditions, including COPD and other ILD. Its short acyl chains lipid A LPS structures interact with Toll-like receptor (TLR) 4 and activate transforming growth factor-β-associated kinase-1 (TAK1) by the MyD88 pathway, resulting in p38 mitogen-activated protein kinase phosphorylation and nuclear factor-κB (NF-κB) activation, which activate transcription of the proinflammatory cytokines like IL-8 in alveolar macrophages [42,43]. It likely plays a role in respiratory immune modulation, possibly contributing to local inflammation by activating Toll-like receptors (TLRs), thereby driving the recruitment of immune cells and exacerbating tissue damage. This mechanism could be key in the pathogenesis of GLILD, where chronic immune activation leads to granulomatous lung disease. Furthermore, its outer membrane antigens have been implicated in autoimmune nephropathies due to molecular mimicry, which could also be implicated in the development of GLILD through autoimmune responses [44]. 

Moreover, *B. vulgatus,* a known pathobiont of inflammatory intestinal disease, has also been shown to activate the NF-κB pathway and induce cytokine gene expression [45] and has also been associated with post-acute COVID-19 syndrome [46]. The presence of this species in GLILD may indicate a link between gut dysbiosis and lung disease via the gut–lung axis, where bacterial translocation or metabolites could drive lung inflammation. Furthermore, as with *H. parainfluenzae*, many *Bacteroides* bacteria are among those that have been confirmed to express molecular mimics associated with promoting inflammatory profiles characteristic of several autoimmune diseases, and increased antigen amounts in a proinflammatory environment could also develop autoreactive responses in GLILD [47]. However, evidence is yet limited, as most studies, even in these other disorders, do not reach species level, and the role of many species within a genus in the ecosystem may significantly vary. We could hypothesize that ecologically keystone bacteria able to promote the activation of the NF-κB pathway or with molecular mimicry could be of importance in the pathophysiology of GLILD.

Furthermore, in the cluster network and correlation analyses, we have identified that GLILD patients have a profound microbiome disruption, with complex and highly interconnected microbial networks across all niches, especially in fecal samples, when compared both to CVID patients without GLILD and healthy controls. The abundance of strong positive and negative correlations in GLILD indicates severe systemic dysbiosis, highlighting significant microbial instability and dysfunction in these patients. This goes in line with previous hypotheses indicating that GLILD is only the lung manifestation of a systemic disease, with association with other comorbidities such as lymphoproliferation or splenomegaly, among others [48,49,50].

Also, regarding these analyses, some bacterial species that show significantly higher abundances in the sputum of GLILD patients compared to other CVID patients or healthy controls are also part of these complex interbacterial networks in the sputum of GLILD patients, while being absent in patients without GLILD or healthy controls. Examples include *L. mirabilis*, *H. parainfluenzae*, *G. elegans*, and *Corynebacterium matruchottii*, which was previously associated with systemic immune dysregulation in CVID (Cabanero-Navalon et al., 2023). Some of these bacteria are also present in salivary networks, along with others, such as *C. gracilis*. Additionally, certain more abundant GLILD-associated fecal species, like an unspecified species of *Ruminococcus* from the torques group and *B. vulgatus*, also appear to function as keystone species in their respective microbial networks without being present in non-GLILD or healthy controls’ microbial networks. This suggests that these bacteria may play a crucial role in maintaining the microbial ecosystem in GLILD patients, potentially contributing to immune dysregulation and chronic inflammation across different body niches, resulting in this potentially systemic disease.

In this study, we aimed to characterize the microbiota associated with GLILD at the species level, providing a holistic view of the microbiome’s role across several body niches. We particularly emphasize the significance of the respiratory tract microbiota in this CVID-associated lung disease, highlighting the previously unknown respiratory niche. However, several important limitations deserve mention. The unicentric nature of the work and the small sample size limit the interpretation of the findings and their generalizability. The influence of prophylactic antibiotic therapy and immunosuppressant treatment could affect microbiome compositions and must be considered. The low relative abundance of some biomarkers could increase the risk of type I error. Moreover, sputum samples are not obtained from bronchoalveolar lavage, which might include salivary contamination. The reproducibility and reliability of the results are potentially enhanced by retaking samples after a few months for comparison, but it was not feasible due to financing constraints. Lastly, this study focuses solely on the bacterial component, omitting the mycobiome and virome, which could also play crucial roles in disease pathogenesis.

## 5. Conclusions

This study provides the first comprehensive analysis of the microbiome in CVID patients with GLILD, highlighting significant differences in saliva, sputum, and fecal samples compared to other CVID subgroups and healthy controls. We identified specific bacterial communities, particularly in the respiratory tract, associated with GLILD, with a non-assigned species of *Conchiformibius* emerging as a potential marker.

Our findings emphasize the importance of the respiratory microbiota in CVID-associated lung disease. However, the single-center nature, small sample size, and influence of prophylactic antibiotics and immunosuppressants limit the generalizability of our results. Future research should explore a more integrative approach, including the mycobiome and virome, and conduct multi-center studies to confirm these findings and develop microbiota-targeted therapies.

## Data Availability

The raw data supporting the conclusions of this article will be made available by the authors upon request.
